# Effects of genetic variants on platelet reactivity and one-year clinical outcomes after percutaneous coronary intervention: A prospective multicentre registry study

**DOI:** 10.1038/s41598-017-18134-y

**Published:** 2018-01-19

**Authors:** Hyung Joon Joo, Sung Gyun Ahn, Jae Hyoung Park, Ji Young Park, Soon Jun Hong, Seok-Yeon Kim, WoongGil Choi, HyeonCheol Gwon, Young-Hyo Lim, Weon Kim, Woong Chol Kang, Yun-Hyeong Cho, Yong Hoon Kim, JungHan Yoon, WonYong Shin, Myeong-Ki Hong, Scot Garg, Yangsoo Jang, Do-Sun Lim

**Affiliations:** 1 Department of Cardiology, Cardiovascular Center, Korea University Anam Hospital, Seoul, South Korea; 2 Department of Cardiology, Yonsei University Wonju Severance Christian Hospital, Wonju, South Korea; 3 Department of Clinical Pharmacology and Toxicology, Korea University Anam Hospital, Korea University College of Medicine, Seoul, South Korea; 4 Department of Cardiology, Seoul Medical Center, Seoul, South Korea; 5 Division of Cardiology, Department of Internal Medicine, Konkuk University College of Medicine, Chungju, South Korea; 6 Division of Cardiology, Department of Medicine, Samsung Medical Center, Sungkyunkwan University School of Medicine, Seoul, South Korea; 7 Division of Cardiology, Department of Internal Medicine, Hanyang University College of Medicine, Seoul, South Korea; 8 Department of Internal Medicine, Division of Cardiology, Kyung Hee University Hospital, Kyung Hee University School of Medicine, Seoul, South Korea; 9 Department of Cardiology, Gachon University Gil Medical Center, Incheon, South Korea; 10 Department of Internal Medicine, Seonam University Myongji Hospital, Goyang, South Korea; 11 Division of Cardiology, Department of Internal Medicine, Kangwon National University School of Medicine, Chuncheon City, South Korea; 12 Division of Cardiology, Department of Internal Medicine, Soonchunhyang University Cheonan Hospital, Cheonan, South Korea; 13 Division of Cardiology, Severance Cardiovascular Hospital, Yonsei University College of Medicine, Seoul, South Korea; 14 East Lancashire Hospitals NHS Trust, Blackburn, Lancashire UK

## Abstract

Clopidogrel is the mainstay for antiplatelet treatment after percutaneous coronary intervention (PCI). The relationship of platelet reactivity and genetic polymorphism with clinical outcomes with newer-generation drug-eluting stents is unclear. We analysed 4,587 patients for the most powerful single-nucleotide polymorphisms (CYP2C19, CYP2C9, ABCB1, PON1, and P2Y12) related to on-treatment platelet reactivity (OPR). The optimal cut-off value of high OPR for major adverse thrombotic events was 266. CYP2C19 was significantly associated with high OPR and the number of CYP2C19*R (*2 or *3) alleles was proportional to the increased risk of high OPR. Death, myocardial infarction (MI), stroke, stent thrombosis, and bleeding events were assessed during a 1-year follow-up period. Primary endpoints were death and non-fatal MI. The cumulative 1-year incidence of death and stent thrombosis was significantly higher in patients with CYP2C19*2/*2, CYP2C19*2/*3, and CYP2C19*3/*3 (Group 3) than in patients with CYP2C19*1/*1 (Group 1). Multivariate Cox proportional hazard model showed that cardiac death risk was significantly higher in Group 3 than in Group 1 (hazard ratio 2.69, 95% confidence interval 1.154–6.263, p = 0.022). No association was reported between bleeding and OPR. Thus, CYP2C19 may exert a significant impact on the prognosis of PCI patients even in the era of newer-generation drug-eluting stents.

## Introduction

Strong platelet inhibition using dual antiplatelet therapy (DAPT) has been the mainstay for the prevention of adverse thrombotic events after percutaneous coronary intervention (PCI). DAPT includes the combination of a P2Y12 inhibitor and low-dose aspirin. The newer, more potent P2Y12 inhibitors (prasugrel and ticagrelor) have been shown to reduce recurrent ischemic events, especially in patients with acute coronary syndrome (ACS), but adverse bleeding events have been a concern. Current guidelines prefer prasugrel or ticagrelor for ACS patients undergoing PCI and recommend clopidogrel in the non-ACS setting^1,2^. However, the concerns related to increased cost, adverse bleeding events, older age, and other comorbidity may limit the use of ticagrelor or prasugrel. Hence, clopidogrel remains the most widely used treatment regimen for PCI.

It is interesting that the East Asian population show different laboratory and clinical thrombogenicity and bleeding characteristics as compared with the Western populations^3^. The “East Asian paradox” refers to the increased prevalence of high on-treatment platelet reactivity (OPR) but similar or lower thrombotic event rates after PCI in East Asian patients and has raised questions over the optimal antiplatelet strategy for the East Asian patients^4^. The proposed mechanism underlying these phenomena includes differences in genetic predisposition such as the higher prevalence of CYP2C19 loss-of-function alleles, which has been observed in East Asian patients^5^. These data indicate that the discrepancy between thrombogenicity characteristics and genotyping may affect clinical outcomes after PCI. A recent meta-analysis has demonstrated that CYP2C19 genotype may contribute to worse cardiovascular outcomes in the Asian population as compared with the Western population, particularly after PCI^6^.

The present study examined the single nucleotide polymorphisms (SNPs) of five genes (CYP2C19 as well as CYP2C9, ABCB1, paraoxonase-1 [PON1], and P2Y12), which were reported to be associated with clopidogrel absorption, metabolism, activation, and resistance^7–11^. Although several studies have examined the relationship between OPR as well as genotypes and clinical outcomes in East Asian patients, these studies were limited in size and patient populations^12–14^. Moreover, in the era of newer-generation, drug-eluting stents (DES), it is still unclear whether OPR and its associated genetic polymorphisms may affect clinical outcomes, including both ischemic and bleeding events. The present study, therefore, enrolled approximately 5,000 patients undergoing PCI and determined the relationship between OPR as well as genotypes and the subsequent major adverse events in Korean patients.

## Results

### Baseline characteristics

Baseline characteristics of the study population are presented in Table S1. Briefly, 2,432 (59.97%) patients had index PCI because of ACS and 834 (18.18%) patients had multivessel disease. About two-thirds (4,386; 63.58%) of the implanted stents were second-generation DES with durable polymers. In addition, 2,234 (32.39%) stents were third-generation DES with biodegradable polymers. Bare metal stents and first-generation DES were rarely used. Mean P2Y12 reaction unit (PRU) with DAPT was 213.88 ± 76.19.

### Defining high on-treatment platelet reactivity (OPR)

To explore the relationship between OPR and clinical outcomes, major adverse thrombotic event (MATE) and bleeding event rates were compared after equally dividing patients into four groups according to their PRU values (Fig. S1). MATE rates were higher in groups with higher PRU values, but no significant difference was observed in the bleeding rate among different groups. Next, we determined the optimal PRU cut-off values for MATE prediction after PCI. The receiver operating characteristic (ROC) curve showed that the area under the curve (AUC) of PRU to predict 1-year MATE was 0.6221 (Fig. S2). The Youden index indicated that the optimal PRU cut-off value was 266. With this cut-off value, the sensitivity and specificity for MATE was 44.00% and 75.25%, respectively. Therefore, a PRU > 266 was defined as high OPR.

### Prevalence of clopidogrel metabolism-related gene variants

Of the five SNPs assessed, PON1 gene variants were the most common (87.97%, Table 1). The prevalence of CYP2C19 loss-of-function alleles (except for CYP2C19*1/*1 and CYP2C19*1/*17) was 62.11% and CYP2C19*1/*2 was the most common (35.10%). The prevalence of the complete loss of a normal allele (CYP2C19*2/*17, CYP2C19*2/*2, CYP2C19*2/*3, CYP2C19*3/*17, and CYP2C19*3/*3) was 14.79%. The prevalence of CYP2C19 gain-of-function alleles (CYP2C19*1/*17, CYP2C19*2/*17, and CYP2C19*3/*17) was only 2.14%, while that of CYP2C9, ABCB1, and P2Y12 gene variants was 7.87%, 59.34%, and 24.06%, respectively.

**Table 1:** Prevalence of genetic variants and their risk analysis for high OPR.

		N (%)	Adjusted OR (95% CI)*	*p*-value
CYP2C19	*1/*1	1682 (36.7)	Reference	
	*1/*17	56 (1.2)	1.71 (0.906–3.245)	0.10
	*1/*2	1610 (35.1)	2.01 (1.675–2.418)	<0.01
	*1/*3	561 (12.2)	2.31 (1.818–2.933)	<0.01
	*1/*R	**2227 (48.6)**	**2.08 (1.749–2.466)**	**<0.01**
	*2/*17	33 (0.7)	2.43 (1.094–5.396)	0.03
	*2/*2	342 (7.5)	3.86 (2.943–5.062)	<0.01
	*2/*3	245 (5.3)	4.43 (3.265–6.022)	<0.01
	*3/*17	9 (0.2)	1.90 (0.375–9.646)	0.44
	*3/*3	49 (1.1)	7.59 (4.135–13.935)	<0.01
	*R/*R	**678** (**14**.**8**)	**4**.**14** (**3**.**339–5**.**141**)	<0.01
CYP2C9	*1/*1	4226 (92.1)	Reference	
	*1/*3	356 (7.8)	0.76 (0.574–1.005)	0.80
	*3/*3	5 (0.1)	0.78 (0.083–7.217)	0.90
PON1	RR	552 (12.0)	Reference	
	QR	2172 (47.4)	0.83 (0.659–1.035)	0.08
	QQ	1863 (40.6)	0.90 (0.719–1.136)	0.95
ABCB1	CC	1865 (40.7)	Reference	
	CT	2101 (45.8)	1.05 (0.898–1.220)	0.36
	TT	621 (13.5)	0.95 (0.758–1.189)	0.49
P2Y12	GG	3483 (75.9)	Reference	
	GT	1007 (22.0)	1.00 (0.837–1.183)	0.52
	TT	97 (2.1)	1.19 (0.746–1.900)	0.46

^*^Odd ratio (OR) with 95% confidence interval (CI) was adjusted for age, sex, body mass index, current smoker, diabetes mellitus, prior coronary artery bypass graft, acute coronary syndrome, multivessel involvement, haemoglobin level, and creatinine level.

### Relationship between gene variants and OPR

After adjustment, multivariate analysis indicated that only CYP2C19 gene variants were an independent risk predictor for high OPR. CYP2C19*3/*3 had the highest odd ratio (OR) for high OPR risk (adjusted OR 7.59, 95% confidence interval [CI] 4.135–13.935, p < 0.0001). The number of CYP2C19*R (*2 or*3) alleles proportionally increased the risk of high OPR (*1/*R, adjusted OR 2.08, 95% CI 1.749–2.466, p < 0.0001; *R/*R, adjusted OR 4.14, 95% CI 3.339–5.141, p < 0.0001). Therefore, 15.4% of patients with CYP2C19*1/*1 and 42.5% of patients with CYP2C19*R/*R had a high OPR (Fig. S3). It is interesting that the presence of the gain-of-function allele (CYP2C19*17) had not significant effect on OPR (Fig. S4). No significant difference was observed in the average PRU values between patients with or without CYP2C19*17. The prevalence of high OPR was the lowest in patients with CYP2C19*17.

### Relation between CYP2C19 polymorphism and clinical outcome

The gain-of-function allele CYP2C19*17 may be a confounder; hence, patients with CYP2C19*17 were excluded, while the remaining patients were categorised into three groups: Group 1, patients with CYP2C19*1/*1, n = 1,682; Group 2, patients with CYP2C19*1/*R (R represent 2 or 3), n = 2,171; and Group 3, patients with CYP2C19*R/*R, n = 636. Baseline characteristics and procedural details of each group are shown in Table 2. As expected, the average PRU of Group 2 was higher than that of Group 1 and lower than that of Group 3. The proportion of patients who had already undergone coronary artery bypass graft was significantly lower in Group 3 (p = 0.028). Furthermore, the average serum creatinine level was significantly higher in Group 3 (p < 0.001). However, no significant difference was observed in the baseline characteristics among the three groups.

**Table 2:** Baseline characteristics and procedural details.

	Group 1 (*1/*1) n = 1682	Group 2 (*1/*R) n = 2171	Group 3 (*R/*R) n = 636	p-value
Age (year)	64.4 ± 10.7	64.53 ± 10.67	64.41 ± 10.95	0.93
Men, n (%)	1186 (70.5)	1545 (71.2)	441 (69.3)	0.78
Body mass index (kg/m^2^)	24.65 ± 3.11	24.6 ± 3.14	24.66 ± 3.12	0.86
Current smoker, n (%)	422 (25.1)	564 (26.0)	163 (25.6)	0.83
Hypertension, n (%)	1065 (63.3)	1374 (63.3)	385 (60.5)	0.32
Diabetes mellitus, n (%)	534 (31.8)	733 (33.7)	201 (31.6)	0.67
Hyperlipidaemia, n (%)	621 (36.9)	813 (37.5)	236 (37.2)	0.84
Prior myocardial infarction, n (%)	117 (7.0)	161 (7.4)	40 (6.3)	0.80
Prior PCI, n (%)	239 (14.2)	300 (13.8)	91 (14.3)	0.95
Prior CABG, n (%)	35 (2.1)	34 (1.6)	5 (0.8)	0.03
Prior cerebrovascular accident, n (%)	118 (7.0)	171 (7.9)	41 (6.5)	1.00
**Diagnosis at the index PCI**				
Stable angina, n (%)	682 (40.6)	870 (40.1)	248 (39.0)	0.24
Unstable angina, n (%)	518 (30.8)	691 (31.8)	215 (33.8)	
NSTEMI, n (%)	225 (13.4)	268 (12.3)	79 (12.4)	
STEMI, n (%)	140 (8.3)	194 (8.9)	51 (8.0)	
**Angiographic features**				
Multivessel disease, n (%)	310 (18.4)	390 (18.0)	119 (18.7)	0.99
Left main disease, n (%)	72 (4.3)	84 (3.9)	28 (4.4)	0.90
Left anterior descending artery, n (%)	998 (59.3)	1309 (60.3)	382 (60.1)	0.64
Type B2/C lesion, n (%)	1655 (74.5)	2116 (75.1)	617 (74.5)	0.96
Reference vessel diameter (mm)	2.99 ± 0.53	2.98 ± 0.65	3.04 ± 1.41	0.17
Minimal lumen diameter (mm)	0.62 ± 0.47	0.62 ± 0.44	0.62 ± 0.45	0.95
Diameter stenosis (%)	79.25 ± 17.92	78.89 ± 23.13	79.16 ± 14.65	0.83
Lesion length (mm)	24.7 ± 12.68	25.06 ± 12.97	24.08 ± 11.88	0.17
**Procedural data**				
Bare metal stent, n (%)	2 (0.1)	4 (0.1)	1 (0.1)	0.37
First-generation drug-eluting stent, n (%)	3 (0.1)	7 (0.2)	2 (0.2)	
Second-generation drug-eluting stent, n (%)	1636 (64.5)	2069 (63.6)	590 (61.8)	
Third-generation drug-eluting stent, n (%)	803 (31.7)	1046 (32.1)	330 (34.6)	
Stent diameter (mm)	3.03 ± 0.48	3.02 ± 0.45	3.02 ± 0.43	0.80
Stent length (mm)	24.27 ± 7.99	24.28 ± 8.27	23.92 ± 7.88	0.45
Stent number (/patient)	1.34 ± 0.63	1.31 ± 0.6	1.29 ± 0.58	0.53
**P2Y12 reaction units**	**191**.**21 ± 76**.**98**	**221**.**46 ± 70**.**97**	**249**.**8 ± 71**.**01**	<**0**.**01**
**Other laboratory findings**				
Haemoglobin (g/dL)	13.6 ± 1.89	13.69 ± 1.8	13.66 ± 1.92	0.36
Platelet count (×1,000/µL)	228.02 ± 63.79	228.88 ± 63.83	225.72 ± 64.24	0.55
AST (IUI/L)	32.16 ± 36.16	32.33 ± 40.83	34.34 ± 50.19	0.50
ALT (IUI/L)	26.9 ± 31.56	25.18 ± 19.38	25.9 ± 24.12	0.12
Total cholesterol (mg/dL)	172.7 ± 45.89	173.1 ± 43.98	173.26 ± 45.72	0.95
LDL-C (mg/dL)	103.38 ± 36.68	104.29 ± 36.88	104.99 ± 38.05	0.64
HDL-C (mg/dL)	42.58 ± 11.53	42.6 ± 11.49	42.81 ± 11.3	0.91
Triglyceride (mg/dL)	142.47 ± 105.59	144.21 ± 93.71	139.1 ± 108.24	0.56
Fasting glucose (mg/dL)	128.73 ± 51.81	131.06 ± 51.77	131.73 ± 56.11	0.35
Creatinine (mg/dL)	1.04 ± 0.93	1.04 ± 0.87	1.28 ± 2.09	<0.01
hsCRP (mg/L)	4.64 ± 18.31	3.68 ± 13.09	5.19 ± 25.84	0.24
Left ventricular ejection fraction (%)	58.53 ± 11.08	59.06 ± 11.04	59.15 ± 11.02	0.30
**Discharge medication**				
Aspirin, n (%)	1675 (99.6)	2157 (99.5)	632 (99.5)	0.77
Clopidogrel, n (%)	1647 (97.9)	2131 (98.3)	625 (98.4)	0.34
Proton pump inhibitor, n (%)	244 (14.5)	375 (17.3)	97 (15.3)	0.23
Statin, n (%)	1575 (93.6)	2018 (93.1)	605 (95.3)	0.36
ACE inhibitor, n (%)	472 (28.1)	571 (26.4)	163 (25.7)	0.17
ARB, n (%)	574 (34.2)	746 (34.4)	213 (33.7)	0.93
Beta-blocker, n (%)	1054 (62.7)	1300 (60.0)	402 (63.3)	0.70

Data were presented as n (%) or mean ± standard deviation. Abbreviations: PCI, percutaneous coronary intervention; CABG, coronary artery bypass graft surgery; NSTEMI, non-ST segment elevation myocardial infarction; STEMI, ST segment elevation myocardial infarction; AST, aspartate aminotransferase; ALT, alanine aminotransferase; LDL-C, low-density lipoprotein cholesterol; HDL-C, high-density lipoprotein cholesterol; hsCRP, high-sensitivity C-reactive protein; ACE inhibitor, angiotensin-converting enzyme inhibitor; ARB, angiotensin II receptor blocker. *R represents *2 or *3.

**Figure 1:**
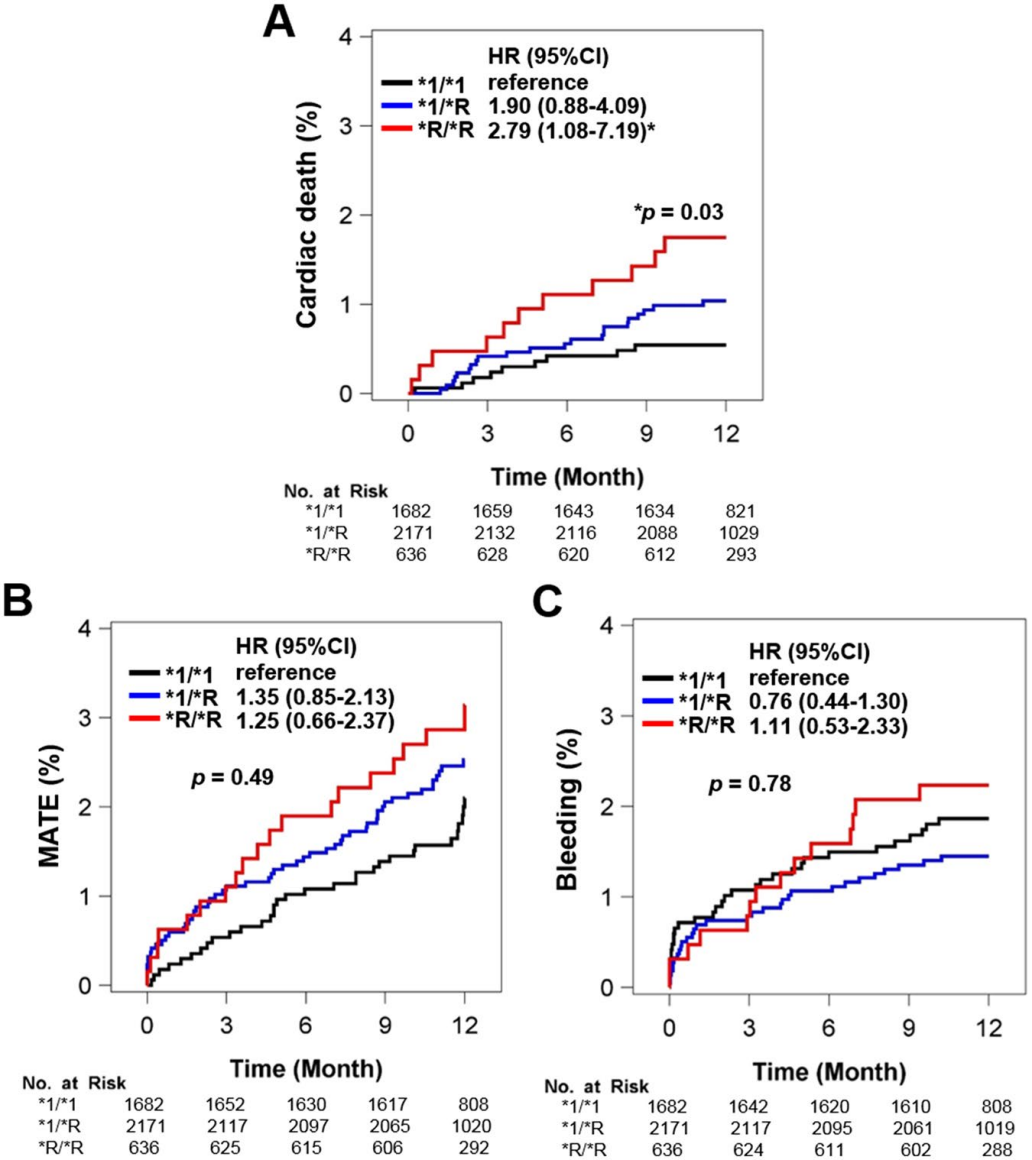
Multivariate Cox proportional hazard model. Cumulative event rates of (**A**) cardiac death, (**B**) major adverse thrombotic event (MATE), and (**C**) bleeding according to CYP2C19 genotype status. *R indicates *2 and *3. HR, hazard ratio; 95% CI, 95% confidence interval.

Overall and grouped in-hospital events and 1-year cumulative rates of clinical events are shown in Table S2 and Table 3, respectively. In the primary endpoints, the cumulative rate of death (all death and cardiac death) was significantly higher in patients with CYP2C19*R/*R (Group 3) as compared to the other groups, but no significant difference was observed in the cumulative rate of non-fatal myocardial infarction (MI) among groups. In the secondary endpoints, the cumulative rate of definite stent thrombosis was significantly higher in Group 3 and no significant difference was reported in the cumulative rates of MATE or bleeding events (defined as Bleeding Academic Research Consortium [BARC] 3/4/5). A multivariate Cox proportional hazard model showed that patients with CYP2C19*R/*R (Group 3) had a higher risk of cardiac death as compared with patients with CYP2C19*1/*1 (Group 1) (adjusted hazard ratio [HR] 2.79, 95% CI 1.08–7.19, p = 0.006, Fig. 1 and Table 4). In addition, old age, low haemoglobin, and low left ventricular ejection fraction were independent predictors for cardiac death.

**Table 3:** In-hospital and 1-year clinical outcome.

	Group 1(*1/*1) n = 1682	Group 2 (*1/*R) n = 2171	Group 3 (*R/*R) n = 636	p-value
**In-hospital mortality, n (%)**	1 (0.1)	0 (0)	2 (0.3)	0.15
**1-year clinical events**				
**Primary endpoints**				
All death, n (%)	25 (1.5)	41 (1.9)	19 (3.0)	0.03
Cardiac death, n (%)	12 (0.7)	30 (1.4)	13 (2.0)	0.01
Non-fatal MI, n (%)	16 (1.0)	25 (1.2)	7 (1.1)	0.64
**Secondary endpoints**				
Stent thrombosis^†^, n (%)	2 (0.1)	9 (0.4)	4 (0.6)	0.04
Ischemic stroke, n (%)	10 (0.6)	11 (0.5)	4 (0.6)	0.96
Repeat revascularisation, n (%)	121 (7.2)	151 (7.0)	42 (6.6)	0.62
MATE^‡^, n (%)	38 (2.3)	68 (3.1)	21 (3.3)	0.10
Bleeding^§^, n (%)	33 (2.0)	38 (1.8)	15 (2.4)	0.74
Any transfusion, n (%)	53 (3.2)	63 (2.9)	24 (3.8)	0.65

*Data were presented as n (%). Abbreviations: MI, myocardial infarction; MATE, major adverse thrombotic event. *R represents *2 or *3.

^†^Stent thrombosis included definite or possible stent thrombosis according to ARC criteria.

^‡^MATE, defined as the composite of cardiac death, non-fatal MI, stent thrombosis, and ischemic stroke.

^§^Bleeding was defined as a clinical bleeding event acceptable for BARC classification type 3, 4, or 5.

**Table 4:** Multivariate Cox proportional hazard model for cardiac death.

Risk factor		Adjusted HR (95% CI)*	*p*-value
CYP2C19	*1/*1	Reference	
	*1/*R	1.92 (0.898–4.085)	0.09
	*R/*R	2.76 (1.112–6.861)	0.03
Age		1.66 (1.1442–2.4138)	0.01
Men		1.42 (0.706–2.854)	0.33
Body mass index		0.79 (0.572–1.0854)	0.15
Smoking		1.74 (0.812–3.73)	0.15
Diabetes		0.67 (0.344–1.318)	0.25
Prior CABG		2.02 (0.266–15.349)	0.50
Acute coronary syndrome		1.05 (0.552–1.98)	0.89
Multivessel disease		2.02 (0.713–5.739)	0.19
Stent (n ≥ 2)		0.48 (0.193–1.182)	0.11
Haemoglobin		0.43 (0.3043–0.6081)	<0.01
Creatinine		1.09 (0.9046–1.3166)	0.36
LDL cholesterol		1.06 (0.7574–1.4823)	0.74
LV ejection fraction		0.75 (0.578–0.9674)	0.03

*Hazard ratio (HR) with 95% confidence interval (CI).

## Discussion

The main findings from Analysis 1 are as follows: (1) higher OPR was significantly associated with a higher incidence of thrombotic events after PCI; the optimal cut-off value for high OPR was a PRU value of 266; and (2) among five SNPs, only CYP2C19 gene variants was associated with the increased risk of high OPR in a dose-dependent manner. The main findings from Analysis 2 are as follows: (1) the 1-year incidence rate of death (all death and cardiac death) was significantly higher in patients with CYP2C19*R/*R (Group 3) as compared to other groups; (2) the incidence of stent thrombosis was higher in patients with CYP2C19*R/*R; and (3) CYP2C19*R/*R was an independent risk predictor for cardiac death.

The present study proposed that a PRU value of 266 was the optimal cut-off value for high OPR for MATE (Fig. S2). OPR has been associated with adverse cardiovascular events after PCI^15^. The recent large ADAPT-DES trial demonstrated that high OPR was not only an independent risk predictor for ischemic events but also a protective factor for bleeding events after DES implantation^16^. In addition, the collaborative analysis of 17 recent trials demonstrated that OPR was related with higher risk of stent thrombosis, bleeding, and subsequent mortality^17,18^. In comparison with the Western patients, the East Asian patients showed different platelet reactivity profiles with a higher prevalence of high OPR, suggestive of the higher cut-off values to define high OPR (252–274 versus 208–240 PRU)^3,19,20^. It is interesting that the East Asian patients, in spite of their higher OPR, showed similar or lower ischemic event risk as compared with the Western patients; this phenomenon is called as the “East Asian Paradox”.

The present study also showed that low OPR by clopidogrel was insufficient to cause a clinically significant bleeding event. Some controversies exist between OPR and bleeding risk. The previous ADAPT-DES trial demonstrated that low OPR increased the risk of bleeding event in US and European patients^16^. On the other hand, Liang *et al*. showed that OPR has no association with bleeding events in 1,016 Chinese ACS patients who took clopidogrel after DES implantation^21^. In recent years, concerns have been raised that the newer P2Y12 inhibitors may contribute to increased bleeding risk, especially in East Asian patients. With this concept in mind, our data may reflect the safety of clopidogrel in preventing bleeding events in East Asian patients.

The platelet function test (VerifyNow P2Y12 assay) and genotype test (CYP2C19 SNPs) were significantly associated with clinical outcomes in the present study. Clopidogrel resistance may be measured by platelet function test, but its reliability and intra-individual variability are quite questionable. Many factors such as smoking, food intake (i.e. caffeine and alcohol), fasting period, exercise, and circadian rhythm affect platelet functions^22^. On the contrary, genotype is invariant and the genotype test provides reliable genetic information from a very small amount of sample. Therefore, the genotype test, rather than the platelet function test, may offer more reliable information on clopidogrel resistance if a certain set of genotype strongly correlates with clopidogrel resistance.

The present study failed to show the significant relationship between OPR and other four SNPs (CYP2C9, PON1, ABCB1, and P2Y12). PON1 genetic polymorphism has been previously suggested to be associated with clopidogrel activity and its clinical efficacy^8^. P-glycoprotein-encoding MDR1 (ABCB1) genetic polymorphism showed poor clinical outcomes, which were probably mediated through the attenuation of clopidogrel absorption^7,9^. CYP2C9*3 loss-of-function allele, which affects the secondary metabolic step of clopidogrel activation, was related with stent thrombosis^10,23^. P2Y12 platelet ADP receptor G52T SNP was also reported to be associated with clopidogrel resistance^11^. However, most of those data were limited for Western patients^24^, suggestive of the ethnic discrepancy between the East Asian and Western patients, similar to CYP2C19 genetic polymorphism.

The present study clearly showed that only CYP2C19 SNPs had a significant effect on OPR (Table 1). Among CYP2C19 SNPs, the prevalence of CYP2C19*17, reported to be associated with the enhanced platelet response to clopidogrel, was very low (1.2%) and its presence had no significant effect on OPR, similar to the other data from East Asian patients^25^. On the other hand, CYP2C19 loss-of-function alleles (*2 and *3) were significantly associated with poor clinical outcomes, consistent with previous results^7,13,14,23,26,27^. Choi *et al*. recently reported that a CYP2C19 loss-of-function allele affected clinical outcomes only in patients with first-generation DES implants^28^. They failed to show statistical significance in patients with newer-generation DES implantation. However, only one case of stent thrombosis occurred in a non-carrier of CYP2C19 loss-of-function allele as compared to five cases in carriers after implantation of the newer-generation DES. The absolute number of composite events was higher in carriers as compared with non-carriers. Thus, the sample size may be too small to determine the statistical significance as compared with the present study.

The present study has several limitations. First, the VerifyNow test was performed only once in the present study. There were no data available regarding the platelet reactivity at the time of cardiovascular events. Thus, there exists a lack of direct evidence that the higher rate of cardiac death in patients with CYP2C19 genetic variants may be elicited by high OPR. In addition, we cannot rule out the possibility that CYP2C19 polymorphism directly affects cardiac death, independent of clopidogrel metabolism and the related platelet response. Indeed, the present study showed that other genetic polymorphisms of drug-metabolising enzymes had no association with platelet reactivity. Second, the present study analysed the clinical events during a 1-year follow-up period of DAPT. Further long-term investigation may provide insight into the clinical impact of genotype according to clopidogrel maintenance or discontinuation. Third, this study had more than one primary endpoint, including death and non-fatal MI. Death was further categorised into all-cause death and cardiac death. Although the endpoints were predefined, these multiple endpoints may make the study purpose unclear. Thus, the present study produced quite descriptive results.

In conclusion, the present study, which included approximately 5,000 PCI patients, demonstrated that the reduced function of CYP2C19 is related to high OPR, leading to increased thrombotic events in patients on clopidogrel after PCI. After adjustment, two copies of CYP2C19 loss-of-function alleles were found as independent prognosticators for cardiac death. Genotype-directed individualisation of antiplatelet therapy remains to be further investigated for East Asian patients.

## Methods

### Study population

Genotyping influences outcomes of coronary artery stenting (GENIUS) is a prospective, multicentre, observational study, which enrolled 5,000 patients undergoing PCI for coronary artery disease in 20 tertiary hospitals in Korea between February 2012 and July 2014. The consumption of both aspirin (100 mg daily) and clopidogrel (75 mg daily) was recommended for 1 year (3 months minimum) after index PCI. Ticagrelor, prasugrel, warfarin, and other new oral anticoagulants were not used during the follow-up period. The study protocol was approved by the Institutional Review Board at each participating centre. Written informed consent was obtained from each patient at enrolment. The study complied with the Declaration of Helsinki and was registered with ClinicalTrials.gov (number NCT02707445).

**Figure 2:**
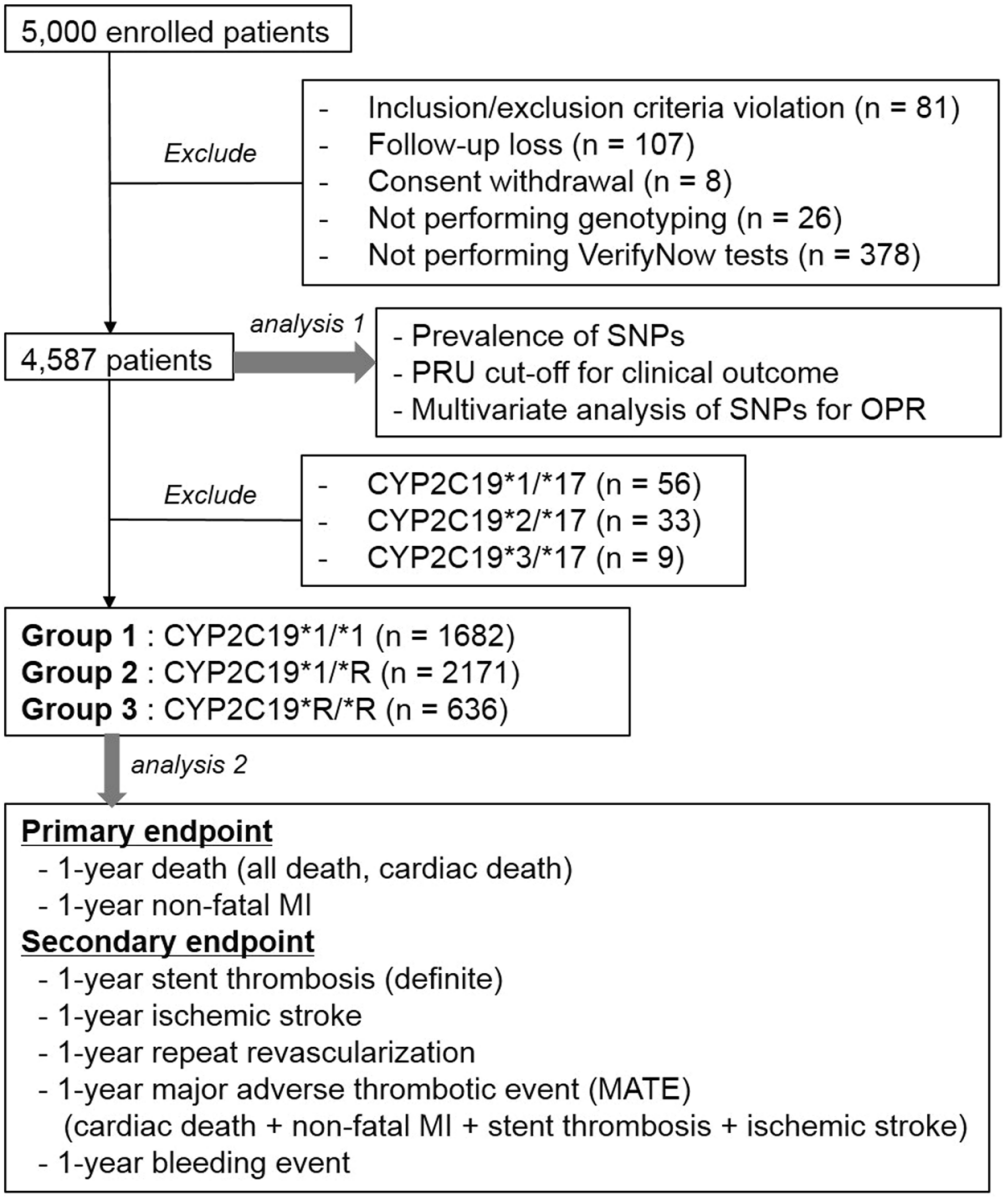
Study scheme. SNP, single nucleotide polymorphism; PRU, P2Y12 reaction unit; OPR, on-treatment platelet reactivity; MI, myocardial infarction.

From the initial cohort of 5,000 patients, 4,587 patients were included in the first analysis (Analysis 1), which screened and selected the most powerful SNPs after excluding the following: inclusion/exclusion criteria violation (n = 81), follow-up loss (n = 107), consent withdrawal (n = 8), missing genotyping results (n = 26), and missing platelet function test results (n = 378). We focused on CYP2C19 genotype and undertook a second analysis (Analysis 2) on the remaining 4,489 patients after excluding the 98 patients with CYP2C19 gain-of-function alleles (Fig. 2).

### Laboratory tests

The inhibitory effect of clopidogrel on platelet reactivity was measured using the VeriyfyNow P2Y12 assay (Accumetrics, San Diego, California, USA). The results were reported as PRU. SNPs measured were CYP2C19*2 (rs4244285), CYP2C19*3 (rs4986893), CYP2C19*17 (rs12248560), CYP2C9*3 (rs1057910), ABCB1 (rs1045642), PON1 (rs662), and P2Y12 (rs6809699). The genotype of each SNP was determined by pyrosequencing using a PSQ 96MA Pyrosequencer (Pyrosequencing AB, Uppsala, Sweden), as previously reported^29^. The residual platelet reactivity and genotype results were blinded to the physicians and patients.

### Definitions

Primary endpoints were death and non-fatal MI. Secondary endpoints were stent thrombosis, ischemic stroke, repeat revascularisation, and bleeding. Stent thrombosis was defined as definite, probable, or possible stent thrombosis based on the Academic Research Consortium Criteria^30^. MATE was considered as a composite endpoint for the ischemic event and defined as the composite of cardiac death, non-fatal MI, ischemic stroke, and stent thrombosis. Bleeding events were categorised according to the definitions of BARC^31^.

### Statistics

Sample size was calculated based on previous trials performed in Korea^27,32^. Assuming a 10% difference among patients with CYP2C19 genetic variants and a 5% drop-out rate, a total of 4,924 patients were required to achieve 95% power to detect statistically significant differences among the groups using the Bonferroni multiple comparison test at a 0.05 significance level. Comparisons between groups were performed using the independent Student’s *t*-test or analysis of variance (ANOVA) test for continuous variables and χ2 test for categorical variables. An ROC curve analysis was used to determine the probability of OPR for MATE. The optimal cut-off value was estimated using the Youden index. The Cox proportional hazard model analyses were performed to compare the incidence of the clinical outcome groups. Multivariable Cox proportional hazard regression model was used to identify risk predictors for MATE and cardiac death. The risk factors were age, sex, body mass index, current smoker, diabetes mellitus, prior coronary artery bypass graft, ACS, multivessel involvement, number of stents, left ventricular ejection fraction, haemoglobin level, creatinine level, and low-density lipoprotein cholesterol level. The results were expressed as HR with a 95% CI and p-value. All tests were two-tailed, and p-values less than 0.05 were considered statistically significant. All statistical analyses were performed using SAS (v9.3, SAS institute Inc., USA).

## Electronic supplementary material

Supplementary Information
