# Supplementary material for: Effects of genetic variants on platelet reactivity and one-year clinical outcomes after percutaneous coronary intervention: A prospective multicentre registry study
Source: Sci Rep. 2018 Jan 19;8:1229. doi: 10.1038/s41598-017-18134-y (PMC5775197; doi:10.1038/s41598-017-18134-y)

Effect of genetic variants on platelet reactivity and one-year clinical outcomes after percutaneous coronary intervention: A Prospective Multicenter Registry Study

Hyung Joon Joo, M.D., Ph.D.[1†], Sung Gyun Ahn, M.D., Ph.D.[2†], Jae Hyoung Park, M.D., Ph.D.[1], Ji Young Park, M.D., Ph.D.[3], Soon Jun Hong, M.D., Ph.D.[1], Seok-Yeon Kim, M.D.[4], WoongGil Choi, M.D.[5], HyeonCheol Gwon, M.D., Ph.D.[6], Young-Hyo Lim, M.D., Ph.D.[7], Weon Kim, M.D., Ph.D.[8], Woong Chol Kang, M.D., Ph.D.[9], Yun-Hyeong Cho, M.D., Ph.D.[10], Yong Hoon Kim, M.D., Ph.D.[11], JungHan Yoon, M.D., Ph.D.[2], WonYong Shin, M.D., Ph.D.[12], Myeong-Ki Hong, M.D., Ph.D.[13], Scot Garg, M.D., Ph.D.[14], Yangsoo Jang, M.D., Ph.D.[13‡], and Do-Sun Lim, M.D., Ph.D.[1‡]

[1]Department of Cardiology, Cardiovascular Center, Korea University Anam Hospital, Seoul, South Korea;
[2]Department of Cardiology, Yonsei University Wonju Severance Christian Hospital, Wonju, South Korea;
[3]Department of Clinical Pharmacology and Toxicology, Korea University Anam Hospital, Korea University College of Medicine, Seoul, South Korea;
[4]Department of Cardiology, Seoul Medical Center, Seoul, South Korea;
[5]Division of Cardiology, Department of Internal Medicine, Konkuk University College of Medicine, Chungju, South Korea;
[6]Division of Cardiology, Department of Medicine, Samsung Medical Center, Sungkyunkwan University School of Medicine, Seoul, South Korea;
[7]Division of Cardiology, Department of Internal Medicine, Hanyang University College of Medicine, Seoul, South Korea;
[8]Department of Internal Medicine, Division of Cardiology, Kyung Hee University Hospital, Kyung Hee University School of Medicine, Seoul, South Korea;
[9]Department of Cardiology, Gachon University Gil Medical Center, Incheon, South Korea;
[10]Department of Internal Medicine, Seonam University Myongji Hospital, Goyang, South Korea;
[11]Division of Cardiology, Department of Internal Medicine, Kangwon National University School of Medicine, Chuncheon City, South Korea;
[12]Division of Cardiology, Department of Internal Medicine, Soonchunhyang University Cheonan Hospital, Cheonan, South Korea;
[13]Division of Cardiology, Severance Cardiovascular Hospital, Yonsei University College of Medicine, Seoul, South Korea;
[14]East Lancashire Hospitals NHS Trust, Blackburn, Lancashire, UK

[†,‡] contributed equally.

Correspondence: Do-Sun Lim, MD, PhD & Yangsoo Jang, MD, PhD

I. Participating institutes, investigators, and trial personnel list of GENIUS study

Participating institutes and investigators;

1. Department of Cardiology, Cardiovascular Center, Korea University Anam Hospital, Seoul, South Korea; Do-Sun Lim, MD, PhD
2. Division of Cardiology, Severance Cardiovascular Hospital, Yonsei University College of Medicine, Seoul, South Korea; Yangsoo Jang, MD, PhD
3. Division of Cardiology, Department of Internal Medicine, Soonchunhyang University Cheonan Hospital, Cheonan, South Korea; WonYong Shin, MD, PhD
4. Department of Cardiology, Yonsei University Wonju Severance Christian Hospital, Wonju, South Korea; JungHan Yoon, MD, PhD
5. Division of Cardiology, Department of Internal Medicine, Kangwon National University School of Medicine, Chuncheon City, South Korea; Yong Hoon Kim, MD, PhD
6. Department of Internal Medicine, Seonam University Myongji Hospital, Goyang, South Korea; Yun-Hyeong Cho, MD, PhD
7. Department of Cardiology, Gachon University Gil Medical Center, Incheon, South Korea; Woong Chol Kang, MD, PhD
8. Department of Internal Medicine, Division of Cardiology, Kyung Hee University Hospital, Kyung Hee University School of Medicine, Seoul, South Korea; Weon Kim, MD, PhD
9. Division of Cardiology, Department of Internal Medicine, Hanyang University College of Medicine, Seoul, South Korea; Young-Hyo Lim, MD, PhD
10. Division of Cardiology, Department of Medicine, Samsung Medical Center, Sungkyunkwan University School of Medicine, Seoul, South Korea; HyeonCheol Gwon, MD, PhD
11. Division of Cardiology, Department of Internal Medicine, Konkuk University College of Medicine, Chungju, South Korea; WoongGil Choi, MD
12. Department of Cardiology, Seoul Medical Center, Seoul, South Korea; Seok-Yeon Kim, MD
13. Department of Cardiology, Chonnam National University Hospital, Gwangju, South Korea; YoungKeun Ahn, MD, PhD
14. Department of Cardiology, Eulji General Hospital, Eulji University College of Medicine, Seoul, South Korea; JaeWoong Choi, MD, PhD
15. Department of Internal Medicine, Gangnam Severance Hospital, Yonsei University College of Medicine, Seoul, South Korea; YoungWon Yoon, MD, PhD

16. Department of Cardiology, Ajou University School of Medicine, Suwon, South Korea; Myoung-Ho Yoon, MD, PhD
17. Division of Cardiology, Department of Internal Medicine, Kyungpook National University School of Medicine, Daegu, South Korea; DongHun Yang, MD, PhD
18. Department of Internal Medicine, Seoul Metropolitan Government Seoul National University Boramae Medical Center, Seoul, South Korea; Jae-Bin Seo, MD, PhD
19. Department of Internal Medicine, Cardiovascular Center, National Medical Center, Seoul, South Korea; SeungMin Choi, MD, PhD
20. Division of Cardiology, Department of Internal Medicine, Yeungnam University Medical Center, Daegu, South Korea; JongSeon Park, MD, PhD

Principal investigator;

Professor Do-Sun Lim, MD, PhD

Department of Cardiology, Cardiovascular Center, Korea University Anam Hospital, Seoul, South Korea

Steering Committee;

1. Do-Sun Lim, MD, PhD, Department of Cardiology, Cardiovascular Center, Korea University Anam Hospital, Seoul, South Korea
2. Yangsoo Jang, MD, PhD, Division of Cardiology, Severance Cardiovascular Hospital, Yonsei University College of Medicine, Seoul, South Korea
3. JungHan Yoon, MD, PhD, Department of Cardiology, Yonsei University Wonju Severance Christian Hospital, Wonju, South Korea
4. Myeong-Ki Hong, MD, PhD, Division of Cardiology, Severance Cardiovascular Hospital, Yonsei University College of Medicine, Seoul, South Korea
5. Weon Kim, MD, PhD, Department of Internal Medicine, Division of Cardiology, Kyung Hee University Hospital, Kyung Hee University School of Medicine, Seoul, South Korea
6. Soon Jun Hong, MD, PhD, Department of Cardiology, Cardiovascular Center, Korea University Anam Hospital, Seoul, South Korea
7. Young-Hyo Lim, MD, PhD, Division of Cardiology, Department of Internal Medicine, Hanyang University College of Medicine, Seoul, South Korea
8. Sung Gyun Ahn, MD, PhD, Department of Cardiology, Yonsei University Wonju

Severance Christian Hospital, Wonju, South Korea

9. Choongki Kim, MD, PhD, Division of Cardiology, Severance Cardiovascular Hospital, Yonsei University College of Medicine, Seoul, South Korea
10. Jae Hyoung Park, MD, PhD, Department of Cardiology, Cardiovascular Center, Korea University Anam Hospital, Seoul, South Korea
11. Hyung Joon Joo, MD, PhD, Department of Cardiology, Cardiovascular Center, Korea University Anam Hospital, Seoul, South Korea

Coordinating Center;

Cardiovascular Research Center, Seoul, South Korea

II. Supplemental Methods

Study protocol

Patients who were aged under 20 years old, pregnant, or had known allergies to aspirin and clopidogrel, were excluded. Patients received a 600 mg loading dose of clopidogrel and aspirin prior to the index PCI, unless they had previously taken these medications. PCI was performed according to standard current guidelines. Heparin was administered during the procedure. Decisions regarding the site of vascular access, type of stent, use of intravascular imaging or use of glycoprotein IIb/IIIa receptor inhibitors were left to the operator's discretion. Clinical events were assessed at 1, 3, 6, 9, and 12 months after the index PCI.

Data management

All data were entered into the electronic case report form, and confirmed by research coordinators at each center. The dropout rates of each follow-up period were less than 4%. All outcomes of interest were adjudicated by a scientific committee from the GENIUS study.

Platelet function test

To reflect a patient's full response to clopidogrel, and to minimize the effect of acute anticoagulation and glycoprotein IIb/IIIa inhibitors used during PCI, it was recommended that a blood sample be obtained, in a sodium citrate bottle, between 1 week and 4 weeks after the index PCI. In case of glycoprotein IIb/IIIa inhibitor use, VeryfyNow P2Y12 assay was performed at least 10 days later.

Genotype test

Briefly, genomic DNA was extracted from peripheral blood mononuclear cells. Five single nucleotide polymorphisms (SNPs) of CYP2C19, CYP2C9, ABCB1, PON1, and P2Y12 genes, which influenced clopidogrel pharmacokinetics, were examined. The SNPs and their sequencing data were validated by comparisons with the direct DNA sequencing of SNPs for randomly selected samples, and the results showed 100% concordance with the present pyrosequencing results, indicating 100% specificity and sensitivity for the method.

Definitions

All deaths were considered cardiac unless there was a clear non-cardiovascular cause. Myocardial infarction was defined by clinically suspicious symptoms or ECG abnormalities together with elevation of cardiac enzymes. Repeat revascularization was defined as any repeat PCI or bypass operation of any coronary vessel.

III. Supplemental Results

Table S1. Baseline characteristics of 4,587 patients

Age (year)	64.46 ± 10.74
Men, n (%)	3234 (70.50)
Body mass index (kg/m^2)	24.62 ± 3.12
Current smoker, n (%)	1166 (25.42)
Hypertension, n (%)	2888 (62.97)
Diabetes mellitus, n (%)	1501 (32.72)
Hyperlipidemia, n (%)	1707 (37.23)
Prior myocardial infarction, n (%)	325 (7.09)
Prior percutaneous coronary intervention, n (%)	640 (13.96)
Prior coronary artery bypass graft surgery, n (%)	77 (1.68)
Prior cerebrovascular accident, n (%)	330 (7.26)
**Diagnosis at the index PCI**	
Stable angina, n (%)	1836 (40.03)
Unstable angina, n (%)	1450 (31.61)
NSTEMI, n (%)	589 (12.84)
STEMI, n (%)	393 (8.57)
**Angiographic features**	
Multivessel disease, n (%)	834 (18.18)
Left main, n (%)	179 (2.92)
Left anterior descending artery, n (%)	2965 (49.33)
Type B2/C lesion, n (%)	4490 (74.94)
Reference vessel diameter (mm)	2.99 ± 0.77
Minimal lumen diameter (mm)	0.62 ± 0.45
Diameter stenosis (%)	79.08 ± 20.13
Lesion length (mm)	24.80 ± 12.78
**Procedural data**	
Bare metal stent, n (%)	7 (0.1)
1st generation drug-eluting stent, n (%)	12 (0.12)
2nd generation drug-eluting stent, n (%)	4386 (63.58)
3rd generation drug-eluting stent, n (%)	2234 (32.39)
Stent diameter (mm)	3.01 ± 0.46
Stent length (mm)	24.22 ± 8.10
Stent number (/patient)	1.32 ± 0.61
**P2Y12 reaction units**	213.88 ± 76.19

**Other laboratory findings**	
Hemoglobin (g/dL)	13.64 ± 1.85
Platelet count (×1000/μL)	228.13 ± 63.76
AST (IUI/L)	32.52 ± 40.36
ALT (IUI/L)	25.90 ± 25.12
Total cholesterol (mg/dL)	172.94 ± 44.93
LDL-cholesterol (mg/dL)	104.04 ± 36.96
HDL-cholesterol (mg/dL)	42.63 ± 11.47
Triglyceride (mg/dL)	142.81 ± 100.18
Fasting glucose (mg/dL)	130.31 ± 52.38
Creatinine (mg/dL)	1.07 ± 1.15
hsCRP (mg/L)	4.24 ± 17.24
Left ventricular ejection fraction (%)	58.88 ± 11.06
**Discharge medication**	
Aspirin, n (%)	4561 (99.52)
Clopidogrel, n (%)	4500 (98.19)
Proton pump inhibitor, n (%)	734 (16.03)
Statin, n (%)	4294 (93.71)
ACE inhibitor, n (%)	1233 (26.92)
ARB, n (%)	1571 (34.32)
Beta-blocker, n (%)	2813 (61.38)

Data were presented as n (%) or mean ± SD. PCI, percutaneous coronary intervention; CABG, coronary artery bypass graft surgery; NSTEMI, non-ST segment elevation myocardial infarction; STEMI, ST segment elevation myocardial infarction; LDL-C, low density lipoprotein cholesterol; HDL-C, high density lipoprotein cholesterol; hsCRP, high sensitivity C-reactive protein; ACE inhibitor, angiotensin-converting-enzyme inhibitor; ARB, angiotensin II receptor blocker.

Table S2. In-hospital and 1-year clinical outcomes after PCI

	N (%)
In-hospital mortality, n (%)	3 (0.07)
**1-year clinical events**	
All death, n (%)	88 (1.92)
Cardiac death, n (%)	58 (1.26)
Non-fatal myocardial infarction, n (%)	49 (1.07)
Stent thrombosis, n (%)	15 (0.33)
Ischemic stroke, n (%)	25 (0.55)
Repeat revascularization, n (%)	318 (6.93)
MATE, n (%)	131 (2.86)
Bleeding, n (%)	88 (1.92)
Any transfusion, n (%)	142 (3.1)

Data were presented as n (%). Stent thrombosis included definite or possible stent thrombosis according to ARC criteria. Major adverse thrombotic event (MATE), defined as the composite of cardiac death, non-fatal MI, stent thrombosis, and ischemic stroke. Bleeding was defined as a clinical bleeding event acceptable for BARC classification type 3, 4, or 5. MI, myocardial infarction.

Figure S1. Relation between OPR and clinical outcomes

(A) Composite major adverse thrombotic event (cardiac death, non-fatal myocardial infarction, stent thrombosis, and ischemic stroke) was associated with quartile distribution of PRU. (B) Bleeding event rate was not associated with quartile distribution of PRU. Q1 indicates PRU 0 to 167; Q2, PRU 168 to 216; Q3, PRU 217 to 264; and Q4, PRU ≥265. PRU, P2Y12 reactive unit; OPR, on-treatment platelet reactivity.

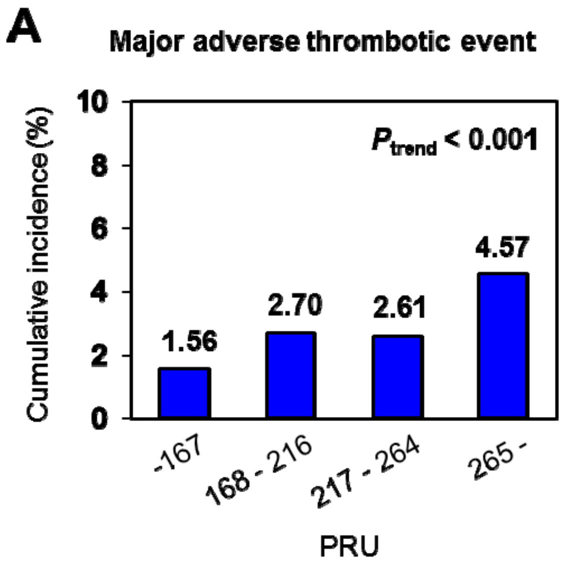

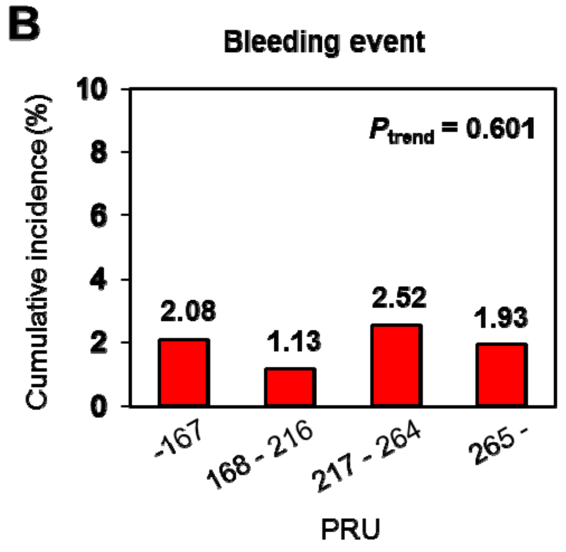

Figure S2. ROC curve of OPR for major adverse thrombotic event

ROC, receiver-operator characteristic; OPR, on-treatment platelet reactivity; AUC, area under the curve.

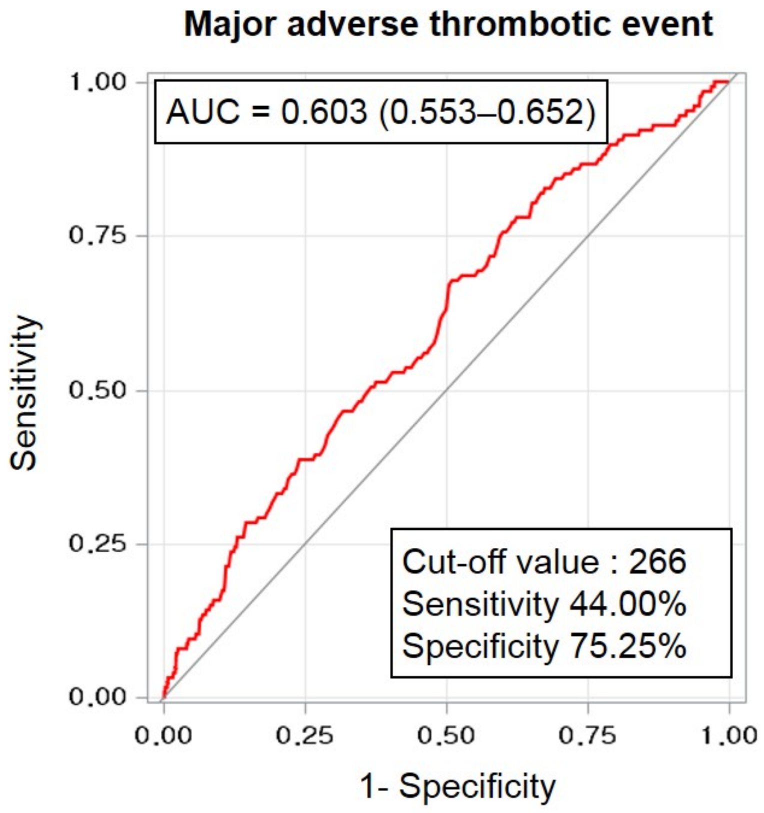

Figure S3. Prevalence of high OPR in CYP2C19 genetic variants

(A) PRU value and (B) Prevalence of high OPR according to CYP2C19 genotype status. High OPR was defined as PRU ≥ 266. *R indicates *2 and *3. PRU, P2Y12 reactive unit; OPR, on-treatment platelet reactivity.

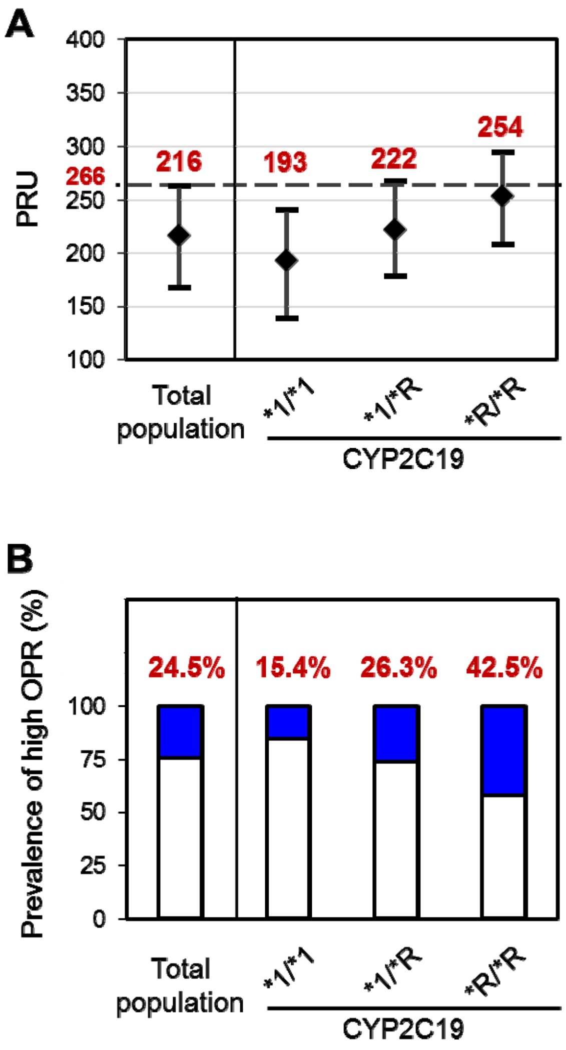

Figure S4. Impact of CYP2C19*17 gene variant on the platelet reactivity

(A) PRU value and (B) Prevalence of high OPR according to CYP2C19 genotype status. High OPR was defined as PRU ≥ 266. PRU, P2Y12 reactive unit; OPR, on-treatment platelet reactivity.

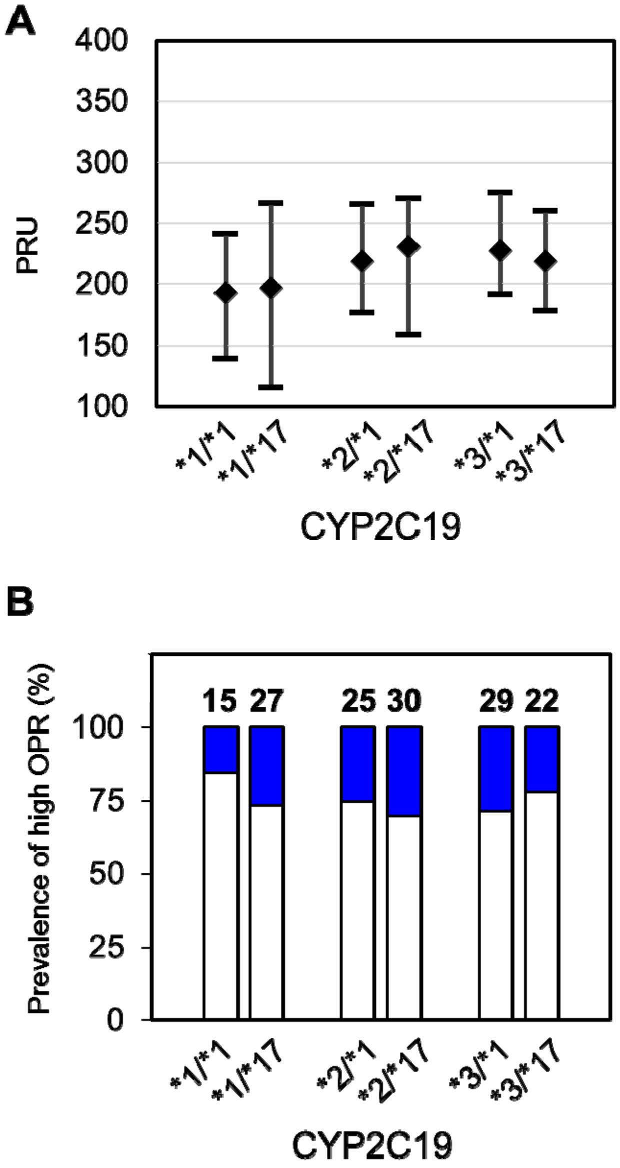

Supplement: Supplementary file 1 — Supplementary Information [file 41598_2017_18134_MOESM1_ESM.pdf]
